# Multimodal sedation guided by processed electroencephalography and autonomic nervous system monitoring for spinal cord stimulator implantation: retrospective identification of anesthetic drug doses

**DOI:** 10.1186/s44158-025-00274-7

**Published:** 2025-08-28

**Authors:** Ashima Suresh, Ana Rita Areal, Maryam Alshemeili, Eric François, Reda Tolba, Francisco A. Lobo

**Affiliations:** 1 Department of Anesthesiology — Integrated Hospital Care Institute, Cleveland Clinic Abu Dhabi, Abu Dhabi, United Arab Emirates; 2 Instituto de Ciências Biomédicas de Abel Salazar, Universidade Do Porto, Porto, Portugal; 3 Department of Pain Medicine — Neurological Institute, Cleveland Clinic Abu Dhabi, Abu Dhabi, United Arab Emirates; 4 Department of Anesthesiology, Yas Clinic Khalifa City/Abu Dhabi Stem Cells Center, Abu Dhabi, United Arab Emirates

**Keywords:** Multimodal sedation, Electroencephalogram, Spinal cord stimulation

## Abstract

**Background:**

Spinal cord stimulation is a validated approach for managing chronic pain syndromes. The stimulator placement typically requires sedation, and an awake phase is needed to ensure optimal lead positioning. We describe a novel multimodal sedation approach using target-controlled infusions of propofol, remifentanil, and dexmedetomidine, combined with boluses of ketamine, guided by electroencephalography and nociception-antinociception balance monitoring.

**Methods:**

This retrospective, single-center cohort study reviewed all spinal cord stimulator procedures, including both trials and permanent implants. A standardized anesthetic protocol, administered by a single anesthesiologist, included target controlled infusions of propofol, remifentanil, and dexmedetomidine, with additional boluses of ketamine. Processed electroencephalogram guided sedation depth, and antinociception was assessed using the Analgesia Nociception Index. Data collected included drug doses, time to intraoperative awakening, hemodynamic stability, and airway management.

**Results:**

A total of 25 procedures (11 trials, 14 permanent implants) were analyzed in 21 patients, with 4 patients undergoing both procedures. All patients received the same four-drug regimen. The median (interquartile range) minimum and maximum effect-site concentrations of propofol required to achieve an adequate level sedation (level − 4 of the Richmond Agitation-Sedation Scale) were 1 (0.5) µg/mL and 1.5 (0.8) µg/mL, respectively. The median (interquartile range) minimum and maximum effect-site remifentanil concentrations needed to achieve sufficient antinociception (Analgesia Nociception Index between 50 and 70) were 0.5 (0.3) ng/mL and 1.2 (0.4) ng/mL, respectively. The median (interquartile range) minimum and maximum effect-site concentrations of dexmedetomidine required to achieve adequate antinociception were 0.3 (0.1) ng/mL and 0.5 (0.1) ng/mL, respectively. The median (interquartile range) dose of ketamine was 25 (20) mg. The ketamine dose used during the implant was significantly higher than during the trial procedure (30 (30) vs. 20 (10) mg), *p* = 0.006. The average time to intraoperative awakening was 114 ± 56 s, and there was no significant difference between the trial and implant groups.

**Conclusions:**

This study demonstrates the feasibility and safety of a multimodal sedation protocol for the placement of a spinal cord stimulator, combining propofol, remifentanil, dexmedetomidine, and ketamine, guided by electroencephalogram and nociception-antinociception monitoring.

## Background

Spinal cord stimulation (SCS) was introduced into clinical practice in 1967 [1] and today has an important role in the treatment of chronic pain [2]. SCS devices send electrical signals through electrodes placed in the epidural space to targeted areas of the spinal cord, such as the dorsal column or the dorsal root ganglion [3]. SCS is indicated for various painful conditions such as failed back surgery syndrome, peripheral neuropathy, intractable radiculopathy, complex regional pain syndrome, peripheral vascular disease, and refractory angina pectoris when conservative measures fail to provide adequate analgesia. Additional off-label indications have been described, including chronic pelvic pain, chronic abdominal pain, and central neuropathic pain [4].

The procedure consists of two steps: an initial temporary trial to determine the patient’s response to the therapy and a second procedure to percutaneously implant the stimulator device if the patient shows significant clinical improvement.

Various anesthetic techniques for this procedure have been described, including sedation, general anesthesia, and epidural anesthesia, using different combinations of hypnotic and analgesic drugs [5–7]. SCS placement under sedation to minimize pain and discomfort is commonly employed [7, 8] with an intraoperative awake phase for spinal cord mapping and optimal electrode placement. General anesthesia has been recommended for patients with morbid obesity, sleep apnea, and anxiety [9].

We aimed to describe and validate a new multimodal sedation approach that combines propofol, dexmedetomidine, remifentanil, and ketamine with dosing guided by electroencephalogram (EEG) and nociception-antinociception balance (NANb) monitoring using heart rate variability (HRV).

## Methodology

### Study design and objectives

This retrospective study aimed to determine the doses of anesthetic drugs in patients undergoing SCS procedures.

The study was conducted in accordance with the Declaration of Helsinki, and the Institutional Research Ethics Committee approved this study (REC CCAD A-2025–012), waiving the requirement for informed consent.

### Population

All cases of SCS-related procedures, including trials and implantations, performed between October 2021 and March 2023 used the same multimodal anesthetic approach guided by processed EEG and NANb monitoring and performed by the same anesthesiologist.

### Data collection

The following data were extracted from electronic medical records (EPIC, Epic Systems Corporation, Verona, WI, USA).

Procedural details are as follows:

- Performance of SCS
- Whether the procedure was a trial or a permanent implant

Data from the anesthetic technique is as follows:

- Propofol (minimum and maximum effect-site (ES) concentrations, µg/ml)
- Remifentanil (minimum and maximum ES concentrations, ng/ml)
- Dexmedetomidine (minimum and maximum ES concentrations, ng/ml)
- Ketamine (total dose, mg)
- Time for intraoperative awakening
- Need of vasopressor drugs
- Need of airways rescue

### Statistical analysis

Data were analyzed using descriptive statistics. The Shapiro–Wilk normality test was performed to assess the normal distribution of continuous variables. Continuous variables are presented as median (interquartile range) or average ± standard deviation, as appropriate. The independent samples Mann–Whitney *U* nonparametric test was used to compare variables between groups. The online statistical software Prism, available at https://www.graphpad.com/features, and the IBM SPSS® Statistics version 30, were used for the data analysis. A *p*-value < 0.05 was considered statistically significant.

### Anesthetic management

All patients had the same anesthetic protocol after a pre-procedural assessment conducted by the same anesthesiologist or by the Center of Perioperative Medicine.

#### Monitoring

All patients received standard monitoring, including electrocardiography, noninvasive blood pressure measurements taken every 3 min, pulse oximetry, and capnography.

Root® platform (Masimo Corporation, Irvine, CA, USA) was used to monitor the electroencephalogram using SedLine® (Masimo Corporation, Irvine, CA, USA). The SedLine® monitor uses a frontal montage in positions F7, F8, Fp1, and Fp2 as specified in the international 10–20 system.

ANI® (M’Doloris Medical Systems, Lille, France) incorporated in the Root® platform was used to assess the NANb. The ANI® is a dimensionless number that ranges from 0 to 100, with values above 70 indicating increased parasympathetic activity and reduced nociception; values below 50 correspond to a dominant sympathetic tone and poor antinociception [10].

#### ,Anesthetic technique

**Fig. 1:**
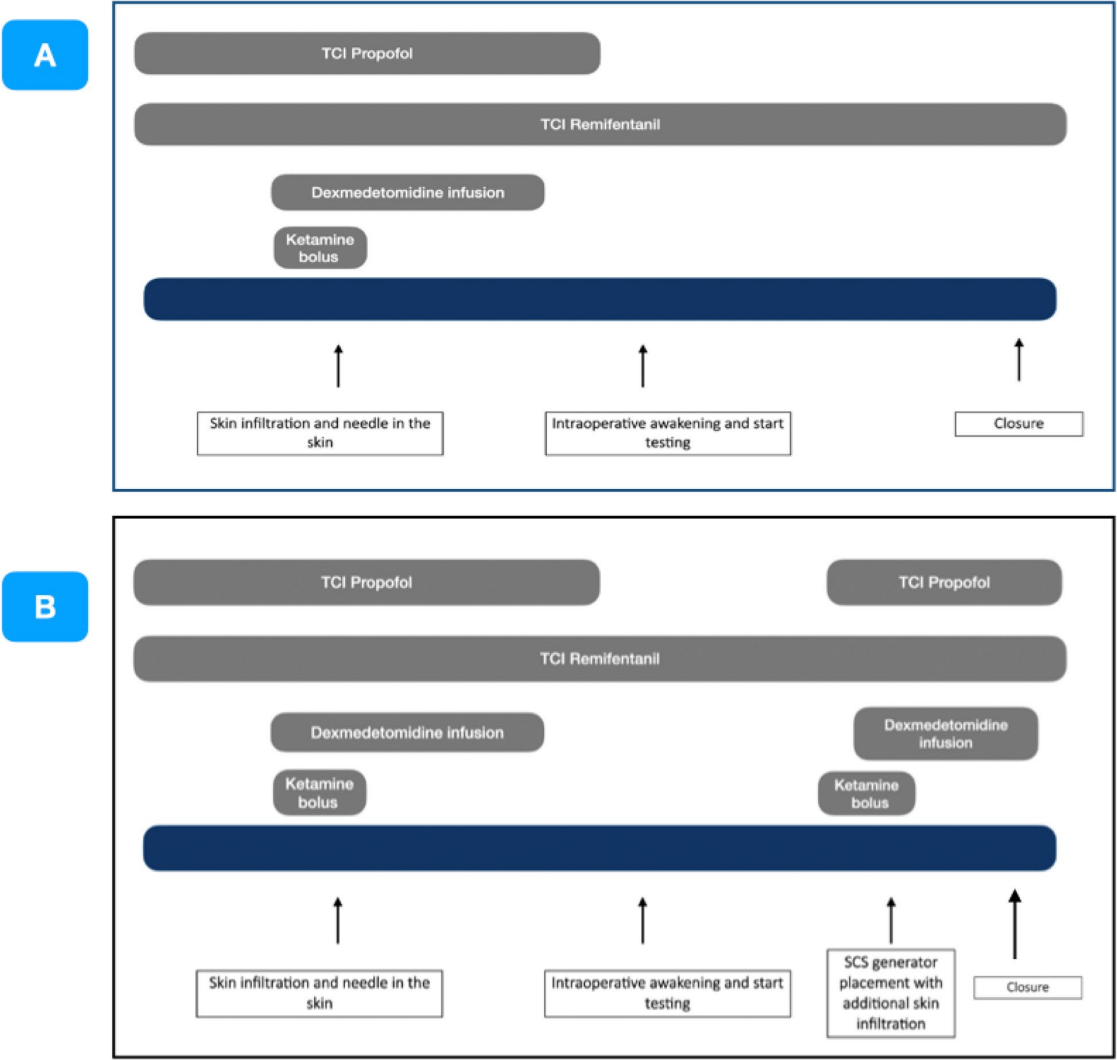
Anesthesia approach timeline. **A** The timeline for anesthetic management for spinal cord stimulator placement (trial procedure). **B** The timeline for anesthetic management for spinal cord stimulator placement (definitive implant procedure)

Sedation was conducted using target controlled infusions (TCI) of propofol, employing the modified Marsh model [11] in ES mode, and remifentanil, utilizing the Minto model [12, 13]. The infusion of dexmedetomidine was managed by TivatrainerX® (Gutta BV, Aerdenhout, the Netherlands, available at https://www.tivatrainer.com) in Assist Mode, using the Hannivoort-Colin model [14, 15] in ES mode (Fig. 1).

**Fig. 2:**
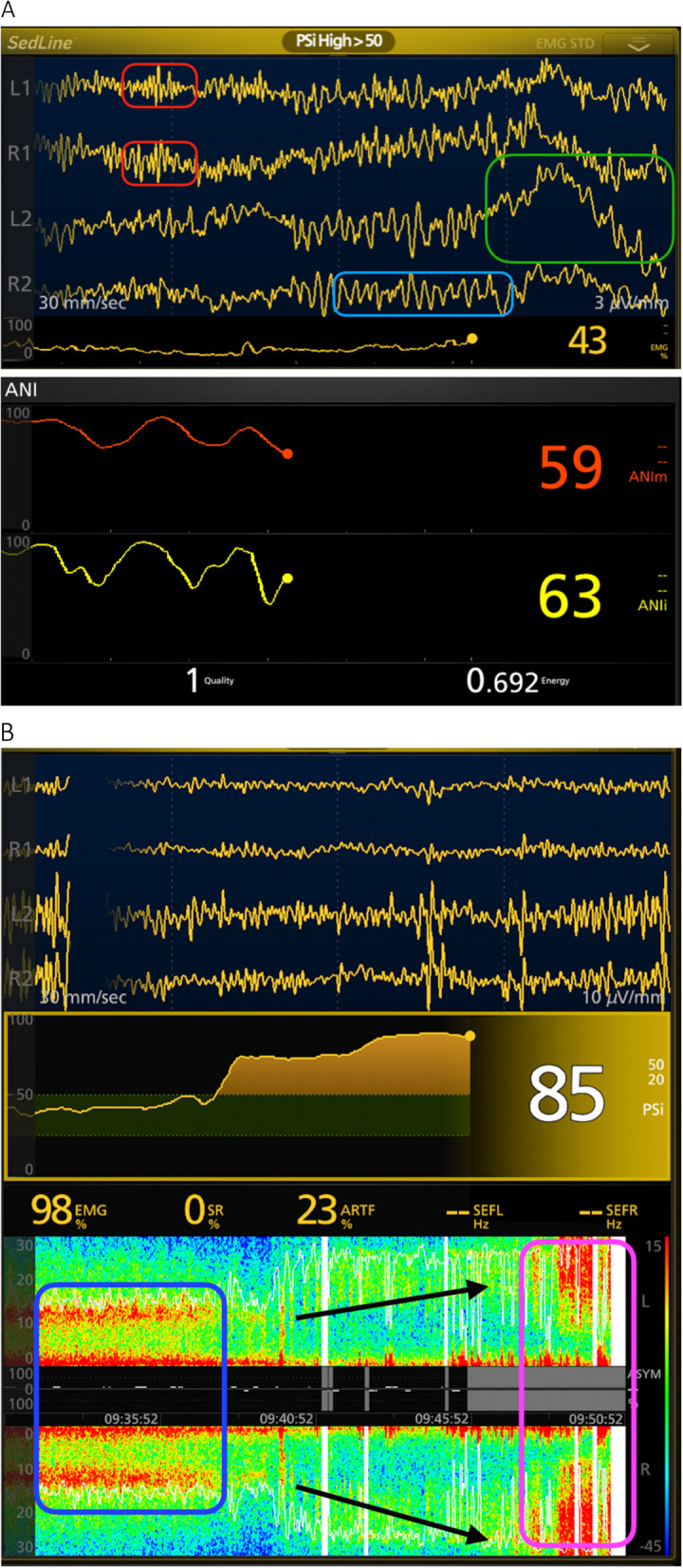
Screenshots of the root monitor displaying examples of EEG and ANI changes during various intraoperative events. **A** The EEG displays a typical pattern of sedation induced by propofol, characterized by alpha rhythms (light blue box) and spindles (red boxes), along with some slow oscillations (green box). The average (orange line) and instantaneous (yellow line) ANI trends indicate oscillations over time corresponding to different NANb levels. **B** Intraoperative awakening displaying a gradual transition from the alpha-delta pattern (blue box) to fast frequencies (pink box)

The changes in the doses of propofol, ketamine, and dexmedetomidine were based on the dynamic changes in their specific electroencephalographic signatures assessed on the raw EEG and on the spectrogram (Fig. 2A), while the doses of remifentanil were based on the ANI.

Propofol TCI started at 1.5 µg/mL in stepwise increments until typical 10-Hz spindles superimposed on delta waves with the peak-max phase-coupling pattern appeared [16], which coincides with a level − 4 of the Richmond Agitation-Sedation Scale [17].

Simultaneously, TCI of remifentanil at 0.5 ng/mL effect site was started with further titration based on the level of nociceptive stimulation and aiming for ANI values between 50 and 70.

After achieving a steady-state concentration of propofol and remifentanil, the infusion of dexmedetomidine was started at a low-nociceptive ES concentration of 0.15 ng/mL, followed by slight increases until the appearance of the typical 12-Hz, short-duration spindles and K complexes generated by dexmedetomidine [18–20]. If such electroencephalographic grapho-elements appear, translating into an unnecessarily high dose of dexmedetomidine, the ES concentration was decreased until the reappearance of the spindles generated by propofol.

Small boluses of 5 mg of ketamine were administered after achieving the steady-state concentration of dexmedetomidine, avoiding decreased alpha power or appearance of faster oscillations [21].

During the induction, paracetamol (1 g IV), parecoxib (40 mg IV), and ondansetron (4 mg IV) were administered.

Concentrations of propofol, remifentanil, and dexmedetomidine were titrated closely to the moment of intra-procedural awakening. Subsequently, the infusions of dexmedetomidine and propofol were interrupted following the proceduralist’s indication regarding the timing for placing the leads and the readiness for neurological testing. TCI of remifentanil was kept during the awake phase.

For the trial procedure, where no more painful events occurred after testing and the SCS generator was placed over the skin, only remifentanil was kept until the end (Fig. 1A).

For the definitive subcutaneous placement of the SCS after testing, the EEG-guided infusions of propofol and dexmedetomidine were resumed and stopped at the end of closure with additional boluses of ketamine and supplementary skin infiltration with local anesthetic drug (Fig. 1B).

The time for intraoperative awakening was defined as the period from when the proceduralist requested the patient to be awake until the patient followed commands and was recorded for each patient using the stopwatch available on the anesthesia monitor.

## Results

Data were retrieved from 25 procedures (11 involved only the placement of the SCS for trial, and 14 had the placement for the definitive implant); 4 patients experienced both procedures, for a total of 21 patients (12 males, 9 females; average age 49 ± 18 years).

All patients received a combination of propofol, dexmedetomidine, ketamine, and remifentanil as part of the multimodal sedation approach described in the methodology.

Spontaneous ventilation was maintained in all patients, with no cases requiring face mask ventilation or any other advanced airway support. No vasopressors were needed to keep the mean arterial pressure above 20% of the baseline values.

The time for intraoperative awakening was 96 (53) s. No significant difference was found between the time for intraoperative awakening during trial seconds and definitive implant second procedures, respectively (96 (57) s vs 106 (52) s with *p* = 0.936) (Table 1).

**Table 1:** Effect-site concentrations of propofol, remifentanil, and dexmedetomidine, doses of ketamine, and time for intraoperative awakening

		**Total**	**Trials**	**Implants**	***p*-value**
Propofol effect-site concentration (µg/mL)	Min	1 (0.5)	0.6 (0.6)	1 (0.4)	0.609
	Max	1.5 (0.8)	1.5 (1.2)	1.6 (0.7)	0.687
Remifentanil effect-site concentration (Ƞg/mL)	Min	0.5 (0.3)	0.5 (0.3)	0.6 (0.3)	> 0.999
	Max	1.2 (0.4)	1.2 (0.5)	1.3 (0.3)	0.344
Dexmedetomidine effect-site concentration (Ƞg/mL)	Min	0.3 (0.1)	0.3 (0.2)	0.3 (0.1)	0.767
	Max	0.5 (0.1)	0.5 (0.2)	0.6 (0.1)	0.075
Ketamine dose	Dose	25 (20)	**20 (10)**	**30 (30)**	**0.006**
Time for intraoperative awakening (s)		96 (53)	96 (57)	106 (52)	0.936

Values as median (interquartile range). Statistically different (*p* < 0.05, Mann–Whitney *U*-test) are marked in bold

### Anesthetic drug doses

The median minimum and maximum ES concentrations of propofol required to achieve an adequate sedative level were 1 (0.5) µg/mL and 1.5 (0.8) µg/mL, respectively. No significant difference was found between minimum and maximum ES concentrations during trials and definitive implant procedures (Table 1).

The median minimum and maximum ES concentrations of remifentanil, calculated by the Minto model, required to achieve an adequate level of antinociception were 0.5 (0.3) ng/mL and 1.2 (0.4) ng/mL, respectively. No significant difference was found between minimum and maximum ES concentrations during trials and definitive implant procedures (Table 1).

The median minimum and maximum ES concentrations of dexmedetomidine, calculated by the Hannivoort model, required to achieve an adequate level of antinociception were 0.3 (0.1) ng/mL and 0.5 (0.1) ng/mL. No significant difference was found between minimum and maximum ES concentrations during trial and definitive implant procedures (Table 1).

The median dose of ketamine was 25 (20) mg. The dose of ketamine used in the definitive implant was significantly higher than the dose used during the trial procedure (30 (30) vs 20 (10) mg) (Table 1).

## Discussion

This study describes a multimodal sedation approach for SCS placement that utilizes TCI of propofol, remifentanil, and dexmedetomidine, supplemented by ketamine boluses, with dosing guidance from frontal EEG and HRV monitoring. The rationale for multimodal sedation is based on the interactions between antinociceptive and hypnotic drugs, which act in different neural pathways through distinct mechanisms. This interaction requires lower doses of the multiple drugs used, resulting in fewer undesirable side effects [22].

Choosing anesthetic drugs with favorable pharmacokinetic/pharmacodynamic (PkPd) profiles and administering them using TCI with adequate models targeting the effect site are crucial for achieving smooth and rapid intraoperative awakening and facilitating faster dose changes compared to those using weight-time-based schemes [23, 24].

Recognizing the specific electroencephalographic signatures of anesthetic drugs enables the titration of the dose to achieve the desired effect and individualize the required dose [25, 26].

A stepwise increase in propofol ES concentration until the appearance of the delta-alpha pattern with peak-max phase coupling [16, 27] was accompanied by impaired arousal and responsiveness. The ES concentration at that moment can serve as the reference target for further anesthetic management.

The initial ES target concentration of 0.5 ng/mL for remifentanil was chosen based on previous data showing the effectiveness of low doses to blunt low-intensity nociceptive stimuli without respiratory depression [28–30] and our preference to use opioid-sparing anesthesia [31–33]. Further titration of remifentanil dosing was based on the ANI. ANI computes signals of heart rate variability, assessing the balance between the sympathetic and parasympathetic activity [10, 34], and has been shown to be helpful as part of a multimodal monitoring in cases of sedation and in conscious patients [35, 36].

Dexmedetomidine has antinociceptive properties by activating inhibitory interneurons located in the dorsal horn of the spinal cord [22, 37, 38] at doses that do not affect the level of arousal, thereby not inducing changes in the EEG but sufficient to be detected by the ANI monitor [35]. Inspecting spindles’ morphology avoids unnecessarily high doses of dexmedetomidine with a high probability of hemodynamic changes [39, 40] and delayed emergence. Spindles generated by dexmedetomidine resemble the sleep spindles that appear during the N2 stage of physiological sleep with maximum power at 13 Hz in frontal regions, while those associated with propofol have an asymmetrical shape with a baseline shift or, alternatively, are superimposed on a slow wave and with two or three waxing and waning oscillations separated by a “breaking point” with a higher frequency component at twice the dominant frequency of the spindle [41, 42].

Dexmedetomidine has been preferred over propofol for SCS placement [8, 43, 44], but no single study has investigated the effectiveness of combining both drugs. Ter Bruggen and colleagues [45] compared the long-term effects of sedation with dexmedetomidine to sedation with propofol during the implantation of SCS and found no statistical difference in long-term outcomes like global perceived effect, pain course, and functional status. However, there was a trend towards greater satisfaction among patients who had sedation with dexmedetomidine.

Combining dexmedetomidine with propofol may significantly decrease the blood pressure and the heart rate [46]. Still, the individualized dosing achieved using TCI with EEG and NANb monitoring may decrease these undesirable side effects.

Ketamine has known peripheral antinociceptive effects [47–50] exerted at low doses which can be determined using a similar approach, based on the absence of changes in the EEG, particularly the appearance of fast beta or gamma oscillations, or, at a lower dose, only a decrease in the power of the alpha frequencies induced by propofol [21]. Due to the significant PkPd variability of ketamine, especially when used with propofol [51], its boluses must be minimal and have an adequate interval between each dose. The higher dose found in the procedure for definitive implant of the SCS reflects the need to supplement antinociception in the last step of the procedure, with the tunnelization of the wires and placing the stimulator subcutaneously.

The fast intraoperative awakening within approximately 2 min, likely due to EEG and NANb monitoring and the PkPd modeling of the IV drugs, enabled us to initiate reliable neurologic testing. We should highlight the smooth intraoperative awakening without agitation or confusion, possibly due to the use of dexmedetomidine [52–54] and the use of the combined use of EEG and ANI [55, 56]; despite a subjective impression, this was the primary advantage noted by the two proceduralists involved in the study, who compared it to their previous experiences with propofol infusion supplemented with fentanyl boluses.

### Limitations

This study has several limitations that must be acknowledged. It is a retrospective cohort study involving a small sample of patients from a single center and under the care of one anesthesiologist, which may introduce sampling bias. However, we have no reason to suspect that our population differs from patients requiring treatment with SCS.

Another limitation of our approach was the inability to use TCI of dexmedetomidine because the institution lacked pumps incorporating PkPd models for dexmedetomidine. However, the use of pharmacokinetic simulation with Tivatrainer® has been described in cases involving intraoperative awakening [29].

Finally, our patient population is relatively young; therefore, we may expect to find lower doses of the anesthetic drugs in older patients [57].

## Conclusions

In summary, we describe a multimodal sedation strategy for placing SCS using propofol, dexmedetomidine, remifentanil, and ketamine, optimized through TCI infusions guided by EEG and NANb monitoring. This study provides average concentrations of these drugs that may serve as a reference in similar clinical cases.

## Data Availability

- Anonymized data will be provided upon a valid request.

## Abbreviations

SCS: Spinal cord stimulation
EEG: Electroencephalogram
NANb: Nociception-antinociception balance
HRV: Heart rate variability
ES: Effect site
TCI: Target controlled infusion
ANI: Analgesia Nociception Index
PkPd: Pharmacokinetic/pharmacodynamic
